# Regulation of adult neurogenesis by GABAergic transmission: signaling beyond GABA_A_-receptors

**DOI:** 10.3389/fncel.2014.00166

**Published:** 2014-06-20

**Authors:** Marta Pallotto, Francine Deprez

**Affiliations:** ^1^Circuit Dynamics and Connectivity Unit, National Institute Neurological Disorders and Stroke, National Institute of HealthBethesda, MD, USA; ^2^Neuroscience Center Zurich, Institute of Pharmacology and Toxicology, University of ZurichZurich, Switzerland

**Keywords:** adult neurogenesis, olfactory bulb, dentate gyrus, GABA_A_ receptor, plasticity

## Abstract

In the adult mammalian brain, neurogenesis occurs in the olfactory bulb (OB) and in the dentate gyrus (DG) of the hippocampus. Several studies have shown that multiple stages of neurogenesis are regulated by GABAergic transmission with precise spatio-temporal selectivity, and involving mechanisms common to both systems or specific only to one. In the subgranular zone (SGZ) of the DG, GABA neurotransmitter, released by a specific population of interneurons, regulates stem cell quiescence and neuronal cell fate decisions. Similarly, in the subventricular zone (SVZ), OB neuroblast production is modulated by ambient GABA. Ambient GABA, acting on extrasynaptic GABA_A_ receptors (GABA_A_R), is also crucial for proper adult-born granule cell (GC) maturation and synaptic integration in the OB as well as in the DG. Throughout adult-born neuron development, various GABA receptors and receptor subunits play specific roles. Previous work has demonstrated that adult-born GCs in both the OB and the DG show a time window of increased plasticity in which adult-born cells are more prone to modification by external stimuli. One mechanism that controls this “critical period” is GABAergic modulation. Indeed, depleting the main phasic GABAergic inputs in adult-born neurons results in dramatic effects, such as reduction of spine density and dendritic branching in adult-born OB GCs. In this review, we systematically compare the role of GABAergic transmission in the regulation of adult neurogenesis between the OB and the hippocampus, focusing on the role of GABA in modulating plasticity and critical periods of adult-born neuron development. Finally, we discuss signaling pathways that might mediate some of the deficits observed upon targeted deletion of postsynaptic GABA_A_Rs in adult-born neurons.

## Adult neurogenesis and GABAergic signaling

Brain development depends on the coordination of numerous processes that go from cell proliferation to circuit refinement. In mature brain circuits, γ-aminobutyric acid (GABA) acts as the main inhibitory neurotransmitter. It is now well known that GABA plays more than a classical inhibitory role and can function as an important developmental signal early in life. Its actions influence processes such as proliferation of neuroblasts and migration, synapse formation, and synapse plasticity. Therefore, GABAergic transmission is essential for proper brain formation and functioning. Imbalance between excitation and inhibition (E/I) due to impaired GABAergic signaling has been implicated in several diseases, such as schizophrenia, epilepsy, autism-spectrum disorders, and intellectual disability. Similarly, GABA exerts a fundamental role in regulating adult neurogenesis, which allows its effects on developing neurons to be studied in adult tissue. The role of GABAergic signaling has been long studied (Bovetti et al., 2011; Berg et al., 2013). Here we will focus on the role of GABA_A_R subunits plays in adult neurogenesis.

In the first part of this review, we will briefly describe the crucial phases that lead a neural stem cell to differentiate and become an adult-born neuron in the dentate gyrus (DG) of the hippocampus and in the olfactory bulb (OB). Then, we will describe the molecular organization of GABA_A_ receptors (GABA_A_R). Finally, we will illustrate the role of GABAergic signals regulating adult neurogenesis. Our goal is to discuss how the spatio-temporal regulation of GABAergic transmission through distinct GABA_A_R subtypes is involved in modulating adult neurogenesis in the OB and DG. In doing so, we will compare the two systems in order to identify common and unique mechanisms mediated by GABAergic transmission.

## Neurogenesis in the adult brain

Adult neurogenesis is the life-long continuous production and functional integration of newborn neurons in the CNS. It represents a process by which the brain can modify itself to face and adapt to external stimuli, as well as to learn and remember. In the rodent brain, adult neurogenesis is restricted to two specific neurogenic niches, the subgranular zone (SGZ) of the DG and the subventricular zone (SVZ) of the lateral ventricles (Gage, 2000; Alvarez-Buylla et al., 2001; Rakic, 2002). The steps of adult neurogenesis include proliferation of stem and progenitor cells, neuroblast fate specification and migration, neuronal differentiation, survival, and integration into the existing circuitry. These steps are under precise spatial and temporal control, but can be modulated by both internal and external stimuli.

**Figure 1:**
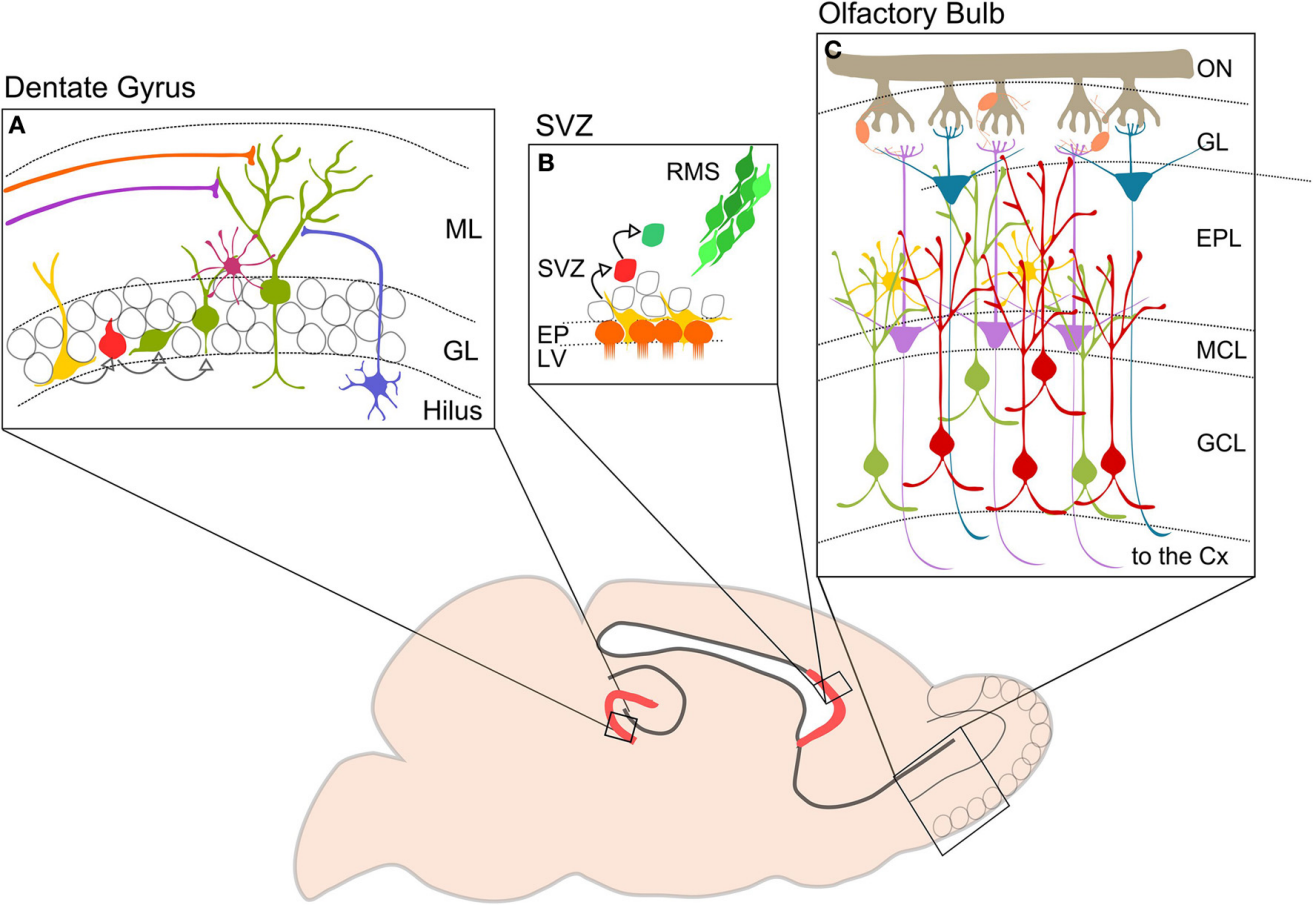
**Neurogenesis in the adult mouse brain**. The picture shows in red the two neurogenerative niches: **(A)** DG of the hippocampus. Type I cells (yellow) divide to generate type II cells (red) that differentiate in immature neurons (green). DG-GCs receive GABAergic inputs from hilar interneurons (blue) and basket cells (purple), and glutamatergic inputs from lateral and medial perforant pathways (orange and violet). **(B)** SVZ. Neurogenic niche contains progenitor or type B cells (yellow) close to the ependymal cells (orange). Type b cells dived slowly and give birth to type C cells (red) which divide again in neuroblasts or type A (green). Neuroblasts migrate in chains along the RMS (from Wang and Kriegstein, 2009 modified). **(C)** Schematic representation showing OB cell types and circuitry. Axons of OSNs are collected in the ON and reach the GL where they make synapses on OB projection neurons: MCs (violet) and TCs (blue). Projection cells are modulated by interneurons, PGCs (orange) located in the GL, and perinatal and adult GCs (green and red) located in the GCL. GCs are also regulated by EPL interneurons (yellow). Axons of MCs and TCs project to higher olfactory cortical centers. ML, molecular layer; GL, granule layer; SVZ, subventricular zone; RMS, rostral migratory stream; EP, ependymal cell layer; LV, lateral ventricle; ON, olfactory nerve; ONS, olfactory sensory neuron; GL, glomerular layer; EPL, external plexiform layer; MCL, mitral cell layer; GCL, granular cell layer; CX, cortex.

In the SVZ three different types of neural precursor cells have been identified: type B radial glia-like progenitors, type C transient amplifying cells and type A migrating neuroblasts. Type B cells divide slowly and give rise to transient amplifying cells and oligodendrocytes. Type C cells divide rapidly and give rise to migrating neuroblasts (Abrous et al., 2005). Neuroblasts exhibit an elongated cell body and have two radially opposed processes (Lois and Alvarez-Buylla, 1993). Before differentiating into olfactory GCs and periglomerular cells (PGCs), these neuroblasts have to migrate for a long distance through the rostral migratory stream (RMS) toward the OB (Figure 1B). During migration, they form a chain consisting of a group of 30–40 cells. After about 5 days, neuroblasts reach the OB, detach from the chain in the RMS and start to migrate to reach either the granule cell layer (GCL) or the glomerular layer (GL) (Luskin, 1993; Lois and Alvarez-Buylla, 1994; Lois et al., 1996). Here they form distinct populations of interneurons, mainly located in the GCL (50–75%) and to a lesser extent in the GL (25%) (Luskin, 1993). Their integration into the preexisting circuitry occurs rapidly. One or two days after entry in the GCL, OB-GCs first receive axo-dendritic inputs from local interneurons (short axon cells), mitral cells, tufted cells, and centrifugal fibers. Finally, upon entering the external plexiform layer (EPL), the dendrite starts branching and forms dendro-dendritic reciprocal contacts with mitral and tufted cells (Whitman and Greer, 2007; Panzanelli et al., 2009). PGCs rapidly migrate toward the GL, send their axon into one or several glomeruli and receive inputs from the olfactory sensory nerve and dendro-dendritic contacts from mitral and tufted cells (Shao et al., 2009). While OB-GCs are strictly GABAergic, PGC can express various transmitter phenotypes including GABAergic, dopaminergic and glutamatergic, depending on their site of origin in the SGZ/RMS (Figure 1C).

The SGZ of the DG contains radial glia-like quiescent neural stem cells (Type I cells), that undergo symmetric division, or give rise to intermediate progenitor cells (Type II), astrocytes or oligodendrocytes. Postmitotic intermediate progenitors differentiate as neuroblasts, which migrate a short distance into the inner GCL and differentiate as GCs. Dentate GCs receive their main excitatory input from the enthorinal cortex and provide glutamatergic inputs to hippocampal pyramidal neurons and CA3 inhibitory interneurons (Figure 1A).

## Molecular organization of GABA_A_ receptors

Among the numerous factors regulating adult neurogenesis, GABAergic signaling, primarily through GABA_A_Rs, plays a major role. GABA_A_Rs are ligand-gated Cl^−^ channels mediating most of the fast inhibitory action of GABA. GABA_A_Rs are also permeable to HCO^3−^ (Kaila et al., 1992), which decrease the effect of inhibition of the Cl^−^ entry, leading to depolarization. GABA_A_Rs are encoded by a large family of subunit genes, grouped in seven classes according to their sequence homology: α (1–6), β(1–3), γ (1–3), δ, ε, θ, π and ρ (1–3) (Macdonald and Olsen, 1994; Sieghart et al., 1999; Sieghart and Sperk, 2002). Differential assembly of these subunits in pentameric channels results in multiple GABA_A_R subtypes with unique functional and pharmacological properties [for α1 subunit, (Kralic et al., 2006), for α3 (Studler et al., 2005), for α5 (Fritschy et al., 1997)].

GABA_A_R subtypes mediating synaptic GABAergic transmission in mature neurons are composed of two α1, α2, α3 subunits together with two β2 or β3 and one γ2 subunit (Jacob et al., 2008). In contrast, the receptors composed of α4, α5, or α6 subunit variants, along with β subunits and δ or γ2 (located at the extrasynaptic sites) mediate tonic GABAergic transmission through ambient GABA (Kilb et al., 2013).

Receptor properties, such as trafficking or clustering, can be regulated by interactions with scaffold proteins and major signaling complexes. Gephyrin, a phospho-protein, is the main postsynaptic scaffolding protein both for GABAergic and glycinergic synapses. It is essential for stabilization of GABA_A_Rs but also interacts with other postsynaptic proteins, like neuroligins and collybistin (Saiepour et al., 2010; Fritschy et al., 2012). It has been shown that the absence of gephyrin results in the loss of postsynaptic GABA_A_Rs (Essrich et al., 1998; Kneussel et al., 1999). In contrast, knockout mice lacking the α1, α2 or γ2 subunits exhibit loss of gephyrin clusters (Essrich et al., 1998; Kralic et al., 2006; Patrizi et al., 2008; Panzanelli et al., 2011). *In vitro* data demonstrate that the phosphorylation state of gephyrin affects GABAergic synaptic function by regulating cluster size and density (Tyagarajan et al., 2011, 2013). Thus, abolishing the phosphorylation of residue S270 favors the formation of supernumerary synapses in cultured hippocampal neurons (Tyagarajan et al., 2011). However, it is also reported by Levi et al. (2004) that gephyrin is not strictly required for GABA_A_R assembly, suggesting the possibility of a gephyrin- independent mechanism of inhibitory synapse development.

Collybistin, another protein which might influence the dynamic and plasticity of GABA_A_Rs at the surface, was shown to bind gephyrin and Cdc-42, potentially affecting the remodeling of the actin cytoskeleton. Further, collybistin can bind directly to neuroligin 2, suggesting that it plays a role in maintenance of GABA_A_R at the plasma membrane (Poulopoulos et al., 2009; Fritschy et al., 2012). These data, along with single-particle tracking studies show that the presence of synaptic and extrasynaptic GABA_A_Rs on the plasma membrane is highly dynamic and regulated by direct or indirect interactions with postsynaptic scaffolding proteins. This feature to adapt GABAergic transmission to the differentiation of their dendrites and incoming synaptic inputs might be particularly important for developing neurons.

So far, most of the studies describing the role of different GABA_A_R subunits are mostly based on KO mice. Despite the considerable insight into GABA_A_R function gained from the use of KO mice, these model systems nevertheless have certain drawbacks. KO mice for GABA_A_R subunits show compensatory effects that impact the neural circuitry, e.g., increased expression of other subunits (Kralic et al., 2006). Adult neurogenesis is moreover a process that involves maturation, integration of single cell into circuits. Given these constrains, it is important to manipulate adult generated cells independently. Recent developments have met this need with new strategies for labeling and manipulating single cells without affecting the entire circuitry. Those new strategies make use of wild type (WT) or transgenic mice model in which the injection of viral vectors in specific brain areas allows labeling or manipulating of specific cell population. This can be done using different promoters or, in case of adeno-associated viruses AAV, different serotype. Because of their spatial and temporal specificity, these manipulations can be done without affecting the development of the brain. This approach is particularly useful to study adult neurogenesis, and therefore is widely used in the field.

In the adult OB and DG, distinct GABA_A_R subtypes are expressed in various cells types to mediate both phasic and tonic inhibition, with possible functional and pharmacological specificity among distinct circuits. The subunit repertoire of precursor cells and neuroblasts is much less well established. Here, we briefly summarize what is known about their organization in both systems (Table 1).

**Table 1:**
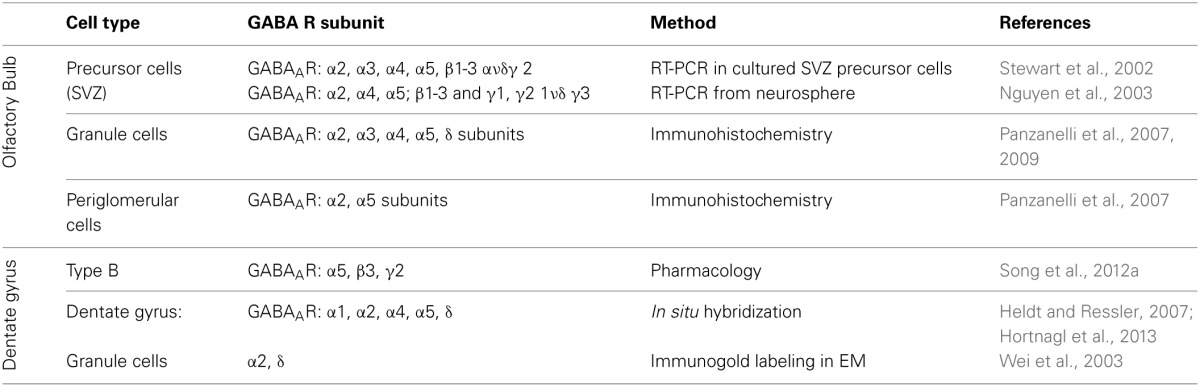
**Distribution of various GABA_A_R subunits in different cell types in olfactory bulb, subventricular zone (SVZ) and dentate gyrus of the hippocampus**.

In the OB, GABAergic GCs express GABA_A_R contain the α2 subunit, and to a lesser extent α3 subunit (Panzanelli et al., 2009), which are responsible for mediating synaptic inhibition. Immunohistochemistry also revealed the presence of extrasynaptic GABA_A_Rs containing the α5 subunit, along with α4 and δ subunits (Panzanelli et al., 2007). GABAergic signaling in PGCs is mainly mediated through synaptic GABA_A_R containing the α2 subunit and extrasynaptically through the α5-GABA_A_Rs (Panzanelli et al., 2007).

Stewart et al. have shown by RT-PCR that precursor cells and neuroblasts in the SVZ express different GABA_A_Rs containing the α2, α3, α4, α5; β1–3 and γ2 subunits and are activated by ambient GABA release (Stewart et al., 2002). Similarly, neurospheres from striatal neuronal progenitors express α2, α4, α5; β1–3 and γ1, γ2 1ν δ γ3 subunits mRNA (Nguyen et al., 2003). Therefore, while adult-born OB-GCs are regulated by both, synaptic and extrasynaptic GABA_A_Rs differing in subunit composition, these subunits are already expressed in OB-GC precursors.

*In situ* hybridization (ISH) and immunohistochemical (IHC) studies have analyzed the GABAergic subunit composition. They have found that in the DG the α2- and α4-GABA_A_R subunits are strongly expressed, while α1 and α5 subunits are moderate expressed in the DG (Heldt and Ressler, 2007; Hortnagl et al., 2013). The GABA_A_R containing the α2 or α1 subunit are responsible for phasic inhibitory transmission of GCs. At extrasynaptic sites, GCs express GABA_A_Rs with the specific subunit combinations α4β2δ and α5β3γ2 (Glykys et al., 2008). In the SGZ neural stem cells respond tonically to GABA via the α5β3γ2 GABA_A_R composition to control their quiescent condition (Song et al., 2012a). Further it has been shown that GABA_A_Rs containing the α4 subunit are expressed in type I cells to control their proliferation rate (Duveau et al., 2011). In conclusion, a huge diversity of GABA_A_Rs is present in the OB and DG, although it is still not clear how the signaling through the different GABA_A_R subtypes is processed. We will address the relevance of distinct GABA_A_R subtypes in modulating critical stages of adult neurogenesis.

Most of the studies described here are performed using RT-PCR or pharmacological approaches combined to KO mice. For most of the GABA_A_R subunits a clear evidence of pattern expression in precursors and neuroblasts is missing. To better understand the role of the different GABA_A_R subunits, a deeper investigation using immunohistochemistry using specific antibodies against the different GABA_A_R subunits and cell markers, would be useful.

## GABA signaling in adult neurogenesis

Adult neurogenesis in the OB and in the DG differs in many aspects. Neuroblasts proliferate in two different niches; they migrate along different routes and for different distances. Then, when neuroblasts incorporate in the preexisting circuitry, they integrate with a very different timing. The functional role of adult neurogenesis, although it is still not entirely clear, is different in the two systems. Nevertheless, GABAergic neurotransmission regulates the entire process of adult neurogenesis in both systems, suggesting common mechanisms, as well as possible differences, including certain functional specificities. In the following paragraph, we aim to compare the role of GABA in regulating adult neurogenesis in the OB and DG, focusing in particular on the contribution of major GABA_A_R subtypes to this process (See Table 2).

**Table 2:**
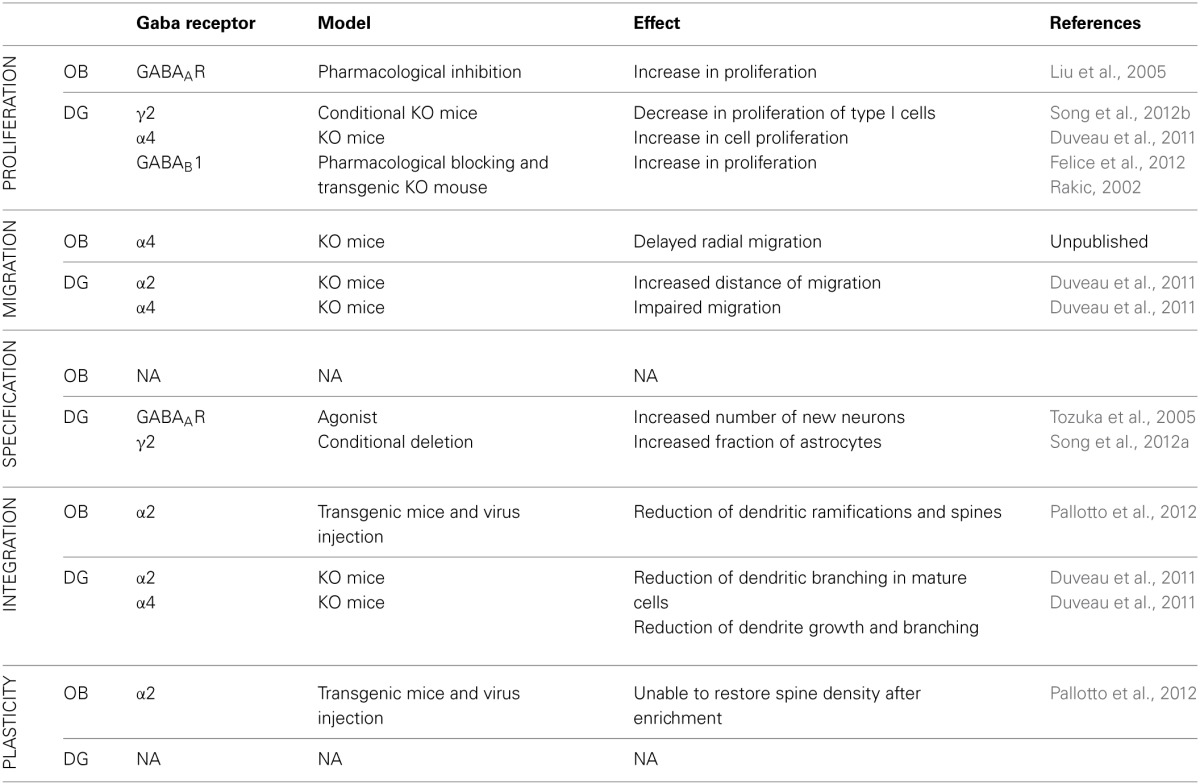
**Comparison of the role of different GABA receptor and receptor subunits in different stages of mouse adult neurogenesis in the OB and DG**.

*NA, not available*.

### Proliferation: GABA is a stop signal in cell proliferation

In the SVZ, precursors and neuroblasts are already sensitive to neurotransmitters (Berg et al., 2013). Neonatal SVZ progenitor cells show chloride currents activated by GABA and muscimol, but they are insensitive to ATP, kainate, NMDA, and ACh (Stewart et al., 2002). The absence of synapses at the EM level suggests that GABA is synthesized and released in a non-synaptic manner by neuroblasts. This conclusion is also supported by electrophysiological and IHC analyses (Doetsch et al., 1997). Moreover, electrophysiological studies have shown that tonic release of GABA activates GABA_A_Rs expressed by neuroblasts and stem cells (Stewart et al., 2002; Wang et al., 2003; Bolteus and Bordey, 2004; Liu et al., 2005). A second source of GABAergic innervation comes from medium spiny or aspiny neurons from the striatum. The activation of striatal neurons increases calcium level into SVZ neuroblasts (Young et al., 2014). Moreover, acute treatment with muscimol decreases BrDU incorporation in type B and C cells of the SVZ (Fernando), and therefore decreases proliferation.

Still it is unknown whether GABA_B_ receptors (GABA_B_Rs) are expressed in the SVZ. The high affinity GABA transporter GAT4 tightly regulates GABA levels in stem cells, but not in neuroblasts. Pharmacological inhibition of GABA_A_R in organotypic cultures *in vivo* induce increased proliferation. Conversely, inhibition of GAT4 produces the opposite effect (Liu et al., 2005).

In the SGZ, type I cells respond to tonic GABAergic stimulation (Tozuka et al., 2005; Ge et al., 2006). In the DG, γ2 subunit (presumably associated with the α5 subunit), has a unique role in maintaining adult precursor cells quiescence. Using a conditional transgenic γ2-KO mouse, Song et al. (2012a,b) found increases in proliferation and increases in symmetrical cell-renewal of type I cells. Interestingly, using an optogenetic approach they identify the source of GABAergic innervation in parvalbumin (PV) positive interneurons, but not somatostatin and vasoactive intestine polypeptide interneurons (Song et al., 2012a). Furthermore, α4 -KO mice showed an increase in proliferation as assessed using BrdU injection (Duveau et al., 2011). A mechanism by which GABA regulates proliferation is through regulation of epigenetic mechanisms that inhibits DNA synthesis (Fernando et al., 2011).

Together these results suggest that at least two distinct GABA_A_R subtypes control stem cells and neural precursor cells.

The role of GABA_B_R in type I cells was investigated by Felice et al. (2012). After blocking GABA_B_R with its antagonist CGP52432, they found an increase in cell proliferation in the ventral hippocampus after 21 days of treatment, but not after acute treatment with the drug. GABA_B_R is made up by different subunits GABA_B_1 and GABA_B_2 (Gassmann and Bettler, 2012). Giachino et al. (2014) demonstrated that GABA_B_1-KO mice show an increased adult progenitor proliferation accompanied with an unaltered cell survival. Since GABA_B_R are expressed not only by GCs, these effects may be a result of an indirect effect.

In summary, GABA_A_R activation decreases the proliferation rate in both OB and DG. GABA concentrations modulate the number of neuroblasts generated by precursor cells, suggesting a negative feedback mechanism to maintain the balance between proliferation and migration (Bordey, 2007 rev).

### Migration: GABA drives neuroblasts migration

OB neuroblasts migrate a long distance, from the SVZ to the OB. They migrate tangentially to the SVZ and once within the OB, they migrate radially respect to the OB using a vascular scaffold (Bovetti et al., 2007). Many factors are involved in this process, and GABA seems to be an important modulator in the tangential migration process. Migrating neuroblasts in the RMS express a variety of GABA_A_Rs in developmently-related combinations (Pathania et al., 2010). Ambient GABA in the RMS reduces the speed of neuroblast migration in acute slices of adult and juvenile mice. Blocking GABA transporters or enhancing GABA release from neuroblasts slows the speed of migration (Bolteus and Bordey, 2004). The migration speed is also regulated by the depolarizing effect of GABA. Mejia-Gervacio et al. (2011) silenced NKCC1 expression with a short harpin RNA strategy in OB acute slices, which did not allow them to make GABA depolarizing. They reported that NKCC1 activity is necessary for maintaining normal migratory speed and regulating the resting membrane potential of postnatal migratory neuroblasts. They also demonstrated that NKCC1 function is strongly reduced at the time in which the cells reach the GCL (Mejia-Gervacio et al., 2011). Conversely, in α4-KO mice, many neuroblasts that complete their tangential migration apparently fail to enter into the GCL, suggesting a role of tonic GABAergic transmission for regulating migration into the gray matter (Fritschy, unpublished).

In the DG, neuroblasts migrate only few microns from the neurogenic niche to their final location, and this process has consequently received less scrutiny. However, Duveau et al. (2011) showed that in α4-KO mice neuroblasts migrate a significantly shorter distance compared to wild type mice. Conversely an opposite phenotype was observed in α2-KO mice, where a higher number of neuroblasts migrate deeper into the GCL. In this example, GABA signaling has a dual and opposite role depending on the receptor it acts on. This suggests that the intracellular pathway activated by the two different receptors may contribute to different functions. Moreover, activation of α4 subunit is critical modulating entry of neuroblasts into the GCL in both the DG and the OB.

### Differentiation

In the OB, it has been estimated that 50–75% of neuroblasts become GABAergic OB-GCs, while the remaining become PGCs. The fate of OB interneurons depends on genetic (Kohwi et al., 2005; Waclaw et al., 2006; Saino-Saito et al., 2007), spatial and temporal factors (for a review see Lledo et al., 2006). The role of GABA_A_R-mediated transmission has yet to be investigated.

GABA is one of the major extrinsic factors regulating differentiation of Type II cells of the DG through GABA_A_R. Indeed, administration of a GABA_A_R agonist significantly increased the number of new neurons labeled with BrdU, while the GABA_A_ antagonist has no effect. Progenitors in the SGZ receive GABAergic, but not glutamatergic inputs. GABA induces the expression of NeuroD, a transcription factor that positively regulates neuronal differentiation toward DG-GCs (Tozuka et al., 2005).

#### Depolarizing action of GABA

One open question that has not been elucidated is the timing of when GABA switches from a depolarizing to a hyperpolarizing agent in adult-born neurons.

Using gramicidin perforated patch clump, Wang et al. (2003) demonstrated that in neuronal progenitors in the SVZ as well as migrating neuroblasts in the SVZ GABA has a depolarizing effect. Mejia-Gervacio et al. (2011) silenced NKCC1 expression with short a harpin RNA strategy in OB acute slices, to make GABA depolarizing. Although these data suggest a depolarizing role for GABA in perinatal neuroblast migration, it is still not clear when GABA switches from a depolarizing to a hyperpolarizing role during OB adult neurogenesis.

In the DG it is well established that GABA has a depolarizing effect on adult-born dentate GCs, recapitulating what happens during the ontogenic development. Ge et al. (2006) used a short harpin RNA strategy to silence NKCC1 activity. They found that the switch from GABA-induced depolarization occurs between 14 and 28 dpi (days post injection). More recently, Chancey et al. (2013) discovered that GABA depolarization is needed for AMPA receptor (AMPAR) synaptic incorporation in developing adult-born GCs.

### Integration and maturation

Many studies have described the development and integration of adult born neurons. Similarly, many reviews have been written to summarize these findings, for an in-depth analysis (see Petreanu and Alvarez-Buylla, 2002; van Praag et al., 2002; Carleton et al., 2003; Lledo et al., 2006; Kelsch et al., 2008, 2010; Dieni et al., 2012; Gu et al., 2013; Platel and Kelsch, 2013). Here, we want to review the role of GABA_A_R and GABA_A_R subunits in modulating integration and maturation of adult born neurons.

#### Structural development of adult-born neurons in the OB

eGFP lentiviral injection into the RMS to birth-date adult-born OB-GCs, were used to described GCs development (Petreanu and Alvarez-Buylla, 2002; Carleton et al., 2003; Pallotto et al., 2012). When neuroblasts arrive into the OB after having migrated along the RMS, they exhibit the typical bipolar morphology of migrating cells. According to Alvarez-Buylla classification, we can distinguish 5 different classes of adult born neurons according to their morphology. Class 1 cells are neuroblasts migrating tangentially toward the OB (observed 2–7 days after virus injection—dpi). Class 2 neurons leave the RMS and migrate radially in the OB (5–7 dpi). Class 3 neurons extend a simple apical dendrite toward the mitral cell layer (MCL) (9–13 dpi). In class 4 neurons, the apical dendrite has crossed the MCL and starts branching within the EPL (11–22 dpi). Finally, class neurons 5 are considered morphologically mature GCs with spiny apical dendrites branched in the EPL (from 15 dpi) (Alvarez-Buylla et al., 2001).

Similarly, adult-born GCs have a unique sequence of electrophysiological maturation. Migratory cells (stages 1 and 2) have membrane properties similar to immature precursors, and do not show spontaneous postsynaptic currents. In contrast, stages 3–5 neurons start to show excitatory and inhibitory postsynaptic currents (Carleton et al., 2003).

Pallotto et al. further described the maturation of adult-born GCs. Dendritic growth and ramification was monitored from 7 to 90 dpi (Pallotto et al., 2012). Sholl analysis on developing adult-born OB-GCs demonstrates that the cells reach a maximum in dendritic branching after 30 dpi, thin value decreasing at 90 dpi. A similar pruning has been shown also for dendritic spines. It has been reported that a maximum dendritic spine density in the EPL at 28 dpi when injecting in the SVZ and at 30 dpi when injecting in the RMS (Whitman and Greer, 2007; Pallotto et al., 2012). Similarly, adult-born PGCs undergo dendritic spine pruning, reaching the maximum spine density between 1 and 3 months post-injection (Livneh and Mizrahi, 2011). These authors suggest that dendritic structure is determined by animal age rather than neuronal age. Altogether these findings demonstrate that the integration of adult-born OB interneurons is a long process and that external factors play a role in shaping the adult morphology of the cell through a pruning mechanism.

Using the Cre-lox system to selectively silence α2-GABA_A_R in virally-transfected cells, Pallotto et al. (2012) investigated the role of phasic GABAergic transmission into adult-born OB-GCs. Inactivation of the α2 subunit gene has detrimental effects on adult-born GCs structural maturation. The authors showed that α2-KO cells have reduced dendritic branching and a number of reduced spines when compared to the WTs. Therefore, the presence of postsynaptic α2-GABA_A_Rs is fundamental for the growth of dendrites and spines observed in WT mice.

#### Synaptic development in adult-born neurons in the OB

Adult-born OB-GCs are rapidly targeted by axon terminals as soon as they reach their final position in the GCL (Whitman and Greer, 2007; Kelsch et al., 2008; Panzanelli et al., 2009). GABAergic and glutamatergic contacts form on the dendrites and cell bodies within 3 dpi in the RMS and on apical dendrites only 1 day later, as shown by IHC quantification of synaptic puncta for inhibitory and excitatory synapse markers. In addition, these contacts are already functional, as shown by whole cell patch clamp recordings (Panzanelli et al., 2009).

Quantification of synaptic inputs onto newborn OB-GCs at early stages of development (3–7 dpi) indicates initially a higher fraction of gephyrin positive puncta then PDS95. At 7 dpi, PDS95 clusters were predominant, suggesting a slight delay in the formation of glutamatergic contacts on newborn GCs. Absence of phasic GABAergic inputs though removal of the α2 subunit led to a marked reduction in spontaneous and evoked inhibitory post-synaptic currents (IPSCs). At the molecular level, the loss of α2 subunit is followed by a strong reduction of its scaffolding protein gephyrin. Presynaptic terminals were not affected and no compensatory effects by α3-GABA_A_Rs, also expressed by OB-GCs, were evident. The reduced synaptic GABAergic function inputs also affected the development of glutamatergic contacts. After deletion of α2 subunit in adult-born GCs, Pallotto et al. (2012) found a reduction of glutamatergic synapses demonstrated by a decrease in spontaneous excitatory postsynaptic currents (EPSCs), and a reduction in the PDS95 positive puncta on the spine head.

#### Development of adult-born neurons in the DG

Within 4 weeks after symmetric division, newborn DG-GCs extend their dendrites into the molecular layer, they first receive slow GABAergic inputs from hilar interneurons and from Ivy cells (Deshpande et al., 2013). Later, they receive numerous glutamatergic inputs from lateral and medial perforant pathways, and lastly perisomatic GABA synapses from various types of basket cells [parvalbumin or cholecystokinin- expressing cells (Katona et al., 1999; Song et al., 2013)], as well as axo-axonic contacts from chandelier cells. Adult-born GC axon projections reach the *stratum lucidum* of the CA3 region as well as the *stratum oriens*, where they form mossy fiber terminals (Esposito et al., 2005; Overstreet Wadiche et al., 2005; Toni et al., 2007; Jessberger et al., 2008; Markwardt and Overstreet-Wadiche, 2008; Zhao et al., 2010). From recent literature it is emerging that another important role of GABA neurotransmission is in the development of DG-GCs. Since GCs express different GABA_A_R subunits, Duveau et al. (2011) dissected the role of α2– and α4-containing GABA_A_R using lentivirus injection in KO mice. At 14 dpi α4-KO mice show a significant reduction of dendritic ramification, whereas the initial growth of dendrites was normal in α2- and δ-KO. At later stages (42 dpi) also α2-KO has a reduced dendritic complexity suggesting that the two different GABA_A_R subunits have different roles in the dendritic tree development. However, it is important to note that DG-GCs also receive phasic inhibition by α1-GABA_A_R (Sun et al., 2004), therefore, the deletion of the α2 subunit may be compensated for, and may not cause a complete loss of GABAergic synaptic inputs onto adult-born GCs.

### Plasticity and critical period

Adult-born GC development described above is not only regulated by an intrinsic program or local signaling molecules. The behavior of the neurons is also affected by their cellular age and by changes in the local environment. The term “critical period” is widely used to describe a specific time window in which neuronal proprieties are particularly prone to modification by external stimuli or experience. Consequences of the critical period are an enhanced morphological and synaptic plasticity that may shape behaviors.

Nissant et al. (2009) demonstrated the tendency of adult-born OB-GCs to undergo long-term potentiation (LTP) after their arrival in the bulb. The ability to undergo LTP faded as newborn neurons matured. LTP is the leading candidate mechanism for memory encoding and the presence of LTP only in a defined “time window” (around 20–30 dpi) suggests that newborn GCs are particularly sensitive to synaptic plasticity (Nissant et al., 2009). External stimuli shape the final morphology of OB-GCs acting on synaptic connectivity. Pallotto et al. (2012) documented that adult-born GCs that were subjected to odor enrichment showed increased spine density. This indicates that by controlling odor exposure during a “critical period,” it is possible to control the level of excitatory drive onto GCs trough principal cell activation. The increase in spine density is most likely due to a stabilization of synaptic turnover rate (Livneh and Mizrahi, 2012).

Varying the degree of sensory inputs to the OB, using olfactory enriched environment or depriving sensory stimuli, Pallotto et al. (2012) found that none of the two treatments caused significant changes in spine density in adult-born OB-GCs lacking the α2-GABA_A_R subunit. This observation indicates that GABAergic synaptic transmission mediated by α2-GABA_A_Rs is required for structural adaptations of adult-born GCs in response to sensory challenges during the phase of dendritic/spine exuberance. GABA neurotransmission may be a candidate to regulate the opening/closing of the critical period in adult neurogenesis in the OB.

Between 28 and 42 days after birth, adult-born DG-GCs show a critical period with enhanced LTP (Ge et al., 2007a; Marin-Burgin et al., 2012). In this time window, they have different mechanisms that make them hypersensitive to stimuli. For example, adult-born GCs have lower activation threshold due to an enhanced E/I balance (Marin-Burgin et al., 2012). Furthermore, adult-born DG-GCs express NR2B NMDA receptor (NMDAR) subunit that appears to be associated with enhanced plasticity (Ge et al., 2007a).

Interestingly, the critical period is preceded by the transition of GABA from excitatory to inhibitory (Ge et al., 2006). Recently, Chancey et al. (2013) thoroughly investigated the role of GABAergic depolarization during the critical period. They found that GABAergic synaptic depolarization enables activation of NMDAR in the absence of AMPAR-mediated transmission *in vitro* as well as *in vivo* after a brief exposure to enriched environment. Therefore, GABAergic depolarization is required and allows excitatory synapse un-silencing that is induced by activity.

### Survival

Both in OB and DG, a massive number of adult-born neurons undergo programmed cell death within a month after birth [specifically, 50% in the OB (Petreanu and Alvarez-Buylla, 2002) and 60–80% in the DG (Cameron and McKay, 2001)]. While deletion of some GABA_A_R subunits affect morphology and plasticity of adult-born cells (see previous paragraphs), it does not explicitly affect survival. This raises the question: is morphological maturation unrelated to survival? According to Pallotto, Duveau and Giachino deletion of different GABA_A_R and GABA_B_R subunits do not affect neuroblasts survival (Rakic, 2002; Duveau et al., 2011; Pallotto et al., 2012). However, up to now, few studies explored properly the role of GABA signaling in the survival of adult-born cells. It would be interesting to use specific markers, like caspase or ssDNA antibodies, to detect the cell death rate in adult-born neurons in OB and DG in mice lacking specific subunits of GABA_A_R.

## Beyond GABAergic signaling

**Figure 2:**
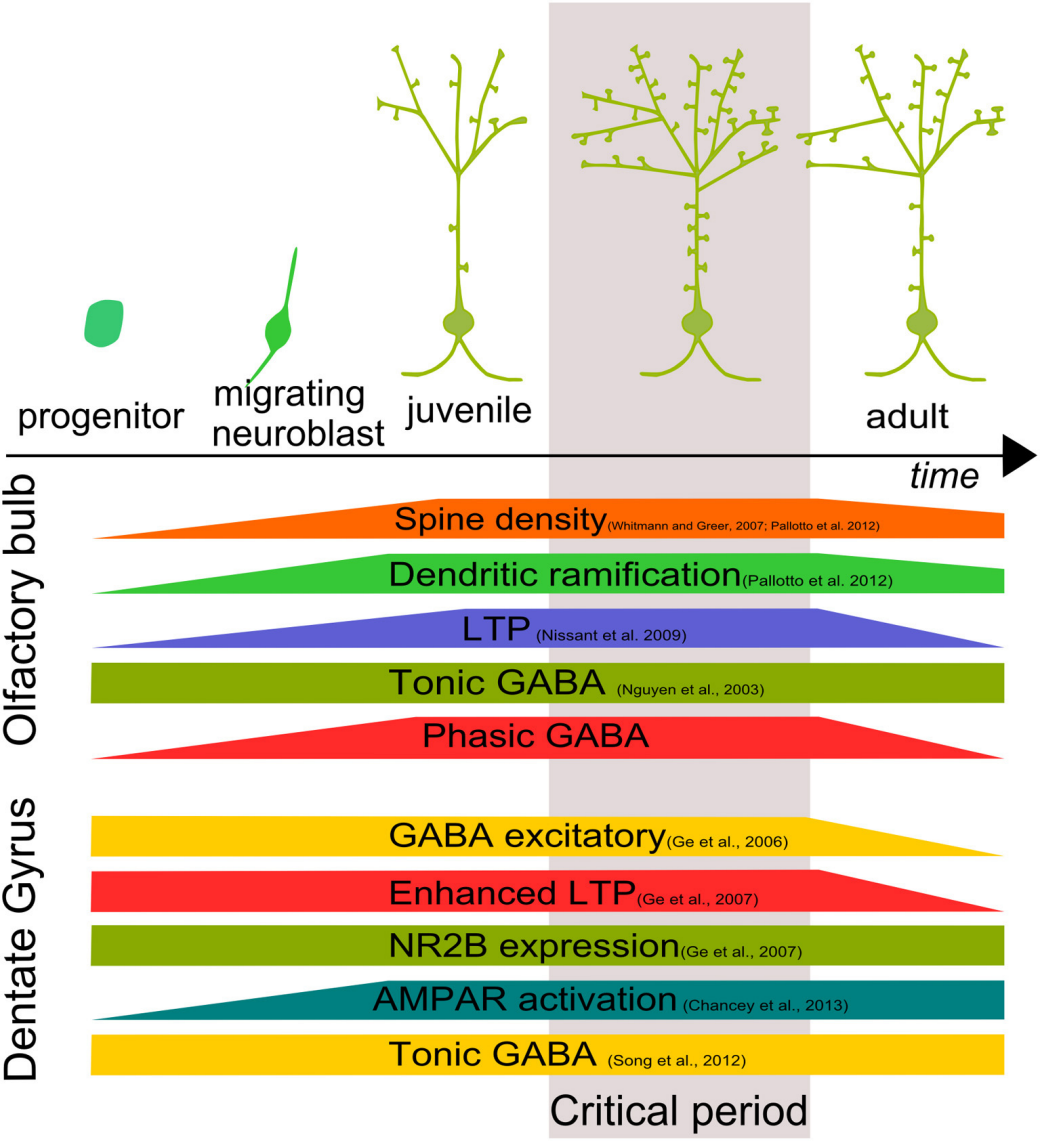
**Development and critical period of adult-born neurons**. Adult–born GC growth is characterized by a time window in which morphological, biochemical and physiological characteristics are finely tuned both in the OB and DG defining a “critical period” for adult-born cell development.

The formation of a functional network in the CNS requires cell integration, synapse formation and maturation through the orchestration of several factors. Understanding these processes is a major challenge in the neuroscience field. Adult neurogenesis is a formidable tool for this purpose. Adult-born cells are rapidly targeted by axon terminals, forming functional excitatory and inhibitory contacts. Considering that during the first month of development, about half of the adult-born cells die (Cameron and McKay, 2001; Winner et al., 2002), synapse formation could represent a way by which GCs are positively selected. Soon after the initial integration step, adult-born GCs go through a period of prominent structural reorganization involving changes in dendritic arborization and spine density (Whitman and Greer, 2007; Livneh and Mizrahi, 2011; Pallotto et al., 2012) (Figure 2).

In both OB and DG, adult-born neurons show a time window in which adult-born neurons are more susceptible to stimuli, showing an enhanced LTP (Figure 2). In the OB, absence of phasic GABAergic input, mediated by α2-GABA_A_Rs, makes adult-born GCs incapable of responding to external stimuli. In the DG, GABAergic activity allows synapse unsilencing driving AMPAR insertion (Chancey et al., 2013). However, the molecular mechanism by which GABA exerts its function is still not clear.

If there is a huge variety of GABA_A_R each of them modulating a different function in adult neurogenesis, then there must be heterogeneous downstream pathways. Here we review three pathways that have been shown to be involved in the downstream molecular signaling. Those pathways may be independent or they may converge and interact at any level.

### Second messengers: examples from CREB and PI3K signaling pathway

Another common mechanism in the OB and DG by which GABAergic transmission affect maturation and the critical period of adult-born neurons could be the cAMP response element-binding protein (CREB) pathway (Merz et al., 2011). CREB is a transcription factor involved in many different aspects of adult neurogenesis (Lonze and Ginty, 2002). CREB and its active form, pCREB is expressed in the SVZ and by adult-born neurons in the OB. pCREB is only sporadically expressed by DCX^+^ neuroblasts in the SVZ, but is strongly present in migrating neuroblasts in the RMS and OB (Herold et al., 2011). The loss of CREB function results in a reduction of the survival rate of newborn neurons and impairs morphological differentiation (Giachino et al., 2005; Herold et al., 2011). Similarly, in the DG the timing of pCREB expression is highly regulated and present in DCX+ cells. In a cell autonomous manner, CREB signaling pathway regulates survival and morphological development (Jagasia et al., 2009).

pCREB controls multiple steps including proliferation, survival, neurite outgrowth and dendrite branching. Formation of spines accompanied with glutamatergic inputs occurs at later stage (28 days after birth), suggesting that GABA might trigger the signaling cascade leading to the phosphorylation of CREB (Magill et al., 2010).

An interesting link between GABAergic activity and CREB signaling come from the work of Jagasia et al. In this work, using an *in vivo* approach with retroviral injection in the DG, the authors demonstrate that CREB phosphorylation and signaling affect survival and maturation and integration of adult born DG-GCs. The peak of CREB phosphorylation occurs when GABA acts with a depolarizing effect on adult born GCs. Using a shNKCC1 virus to ablate the depolarizing effect of GABA, the authors showed that impairment in morphological maturation and differentiation and a reduced survival rate can be rescued by CREB activation (Jagasia et al., 2009).

shRNA against γ2 subunit in adult-born DG-GCs causes a reduced dendritic growth. A similar phenotype is also seen in mice where NKCC1 is down regulated by shRNA (Kim et al., 2012). The selective deletion of the α2 and α4- subunit-containing GABA_A_Rs in adult-born -GCs, alters dendritogenesis but not survival suggesting that there might be another GABA_A_R involved in CREB phosphorylation during the differentiation of adult-born neurons. This different GABA_A_R subunit may regulate the survival but not the development or integration of adult born GCs (Duveau et al., 2011). Despite the lack of strong evidences of GABA_A_R involvement in survival, this phenomenon is not deeply studied. Therefore, we cannot exclude a role of other GABA_A_R subunits having a role in survival of adult-born neurons.

Epigenetic modifications elicited by GABA_A_R activation also influence cell proliferation in the SVZ (Fernando et al., 2011). An interesting paper from Fernando et al. showed *in vitro* and *in vivo* that acute pharmacological modulation of GABA_A_R with muscimol or bicuculline leads to increase or decrease of histone H2AX, respectively. As a consequence, the authors observed a decrease or an increase of BrDU incorporation from type C and B cells of the SVZ. Pharmacological modulation of GABA_A_R, therefore affects proliferation. This effect is also observed long-term after chronic pharmacological treatment, and it affects the number of BrDU positive GCs in the GCL. In a previous paper from the same group, it was demonstrated in embryonic stem cells that GABA_A_R signals through phosphatidylinositol-3-OH kineases to phosphorylate the histone variant H2AX (Andang et al., 2008). Together, these works indicate that GABA_A_R modulate proliferation thought an epigenetic mechanism, that may have important consequence for long-term modulation of the neural niche size and composition, and therefore of the adult neuronal cells that are produced.

### Extrasynaptic receptors mediate their effects via Ca^2+^ signaling

E/I balance has a fundamental role in neuron development. Alterations of the E/I balance cause defects such as impairment of dendritic growth during both in the juvenile and mature development (Cancedda et al., 2007; Ge et al., 2007b). During development, when GABA has an excitatory role, GABAergic-mediated Ca^2+^ signaling mediates several aspects such as migration and maturation. GABA neurotransmitter increase [Ca^2+^_i_] influx thought voltage-sensitive channels (Yuste and Katz, 1991). In cortical development, chemotaxis and chemokinesis are mediated by an increase in intracellular Ca^2+^ concentration stimulated by GABA (Behar et al., 1996). In type A progenitor SVZ cells, GABA_A_R activation induce variation in [Ca^2+^_i_] to modulate proliferation (Nguyen et al., 2003). Similarly, in SVZ-derived neurons, an exposure of 10–30 s to GABA induces [Ca^2+^_i_] increase. GABA_A_R activation is dependent of L-type voltage-gated Ca^2+^ channels (Gascon et al., 2006). In developing DG-GCs, GABA induces Ca^2+^ transients via L-type Ca^2+^ channels. These Ca^2+^ transients are important for suppressing axonal but not dendritic growth (Lee et al., 2012). In the adult brain, spine shrinkage and elimination are promoted by activation of GABA_A_R occurring after an action potential. In this particular case, GABAergic inhibition suppresses local dendritic Ca^2+^ transient that promotes competitive selection of dendritic spines (Hayama et al., 2013). In the DG, GABAergic hippocampal activity depolarizes type-2 cells leading to an increase in intracellular Ca^2+^ concentration and promoting activity-dependent neuronal differentiation. In the SVZ, GABA_A_R induces depolarization leading to the opening of L-type Ca^2+^ channels (Young et al., 2010). In SVZ precursor cells, the presence of tonic currents has been reported (Liu et al., 2005; Bordey, 2007). These data support the notion that Ca^2+^ dynamics are regulated by tonic GABA_A_R activity at early stage of neuronal development; however phasic GABAergic activation cannot be excluded.

### Synaptic receptors and gephyrin signal

GABAergic signals are mediated by both synaptic and extrasynaptic receptors. The presence of synaptic and extrasynaptic GABA_A_Rs on the plasma membrane is a highly dynamic state is and regulated by multiple mechanisms influencing the position and properties of the receptor, including interactions with the gephyrin scaffold. Gephyrin mostly regulates the clustering of synaptic GABA_A_Rs. Gephyrin is crucial for the formation of GABAergic synapses, but it also interacts with other signaling molecules. GABA_A_R-gephyrin interactions regulate gephyrin's clustering properties and/or are anchored at GABAergic synapses by binding to gephyrin. *In vitro* work emphasizes the importance of gephyrin phosphorylation in regulainge GABAergic synaptic function (Tyagarajan and Fritschy, 2010; Tyagarajan et al., 2011, 2013). In particular, inhibiting phosphorylation of the residue Ser270 of gephyrin leads to an upregulation of postsynaptic gephyrin clusters, and consequently to an increase in the frequency and amplitude of mini GABAergic currents (Tyagarajan et al., 2011). Conversely, a mutation in a surface-exposed loop (L2B) prevents gephyrin from clustering and prevents the formation of GABAergic postsynaptic densities (Lardi-Studler et al., 2007). This suggests that the ability of gephyrin in modulating GABAergic synaptogenesis can have a direct influence on the stabilization of GABA_A_Rs in a phosphorylation-dependent manner or on downstream signaling cascades. Downstream gephyrin signaling involves adhesion molecules such as neuroligin 2, or GDP/GTP exchange factors (GEFs) such as collybistin, and also small Rho- GTPases as Cdc-42 or profiling (for a review see Vadodaria and Jessberger, 2013). Cdc-42 can modulate actin and microtubules. This signaling may play a role in maturation and plasticity of adult-born GCs by interacting with the cytoskeleton. Therefore, the morphological deficits seen in adult-born OB-GCs in α2-KO might be due to the fact that gephyrin clusters are disrupted (Pallotto et al., 2012). This presumably leads to a dispersion of signaling molecules such as collybistin and Cdc-42 away from the synapses. In particular, this possibility implies that the reduction of spine density and dendritic growth in adult-born OB-GCs, which has been observed in Pallotto et al. (2012) after selective depletion of α2 subunit, might be due to impaired gephyrin clustering (Pallotto et al., 2012).

To better understand the role of the downstream signal pathway of gephyrin, and therefore its role as scaffold for a proper adult-born cell maturation and integration, it would be interesting to study its different states of phosphorylation and expression patterns.

In summary, GABAergic activity exerts a variety of functions in adult neurogenesis. The role of the neurotransmitter is defined by a variety of GABA_A_R subunits. Sometimes, two different GABA_A_R subunits exert different and opposite effects. For example in the DG, the α2 and α4 subunits have an opposing effect on cell migration (Duveau et al., 2011) (for a comparison see Table 1). Multiple pathways may mediate the specific effects of the different subunits. Here, we have only described a subset of these molecular pathways, which are intermingled, making it difficult to determine their specific roles. For example, it is possible that GABA induces influx of Ca^2+^ in newborn GCs leading to CREB induced gene expression. Similarly, Ca^2+^ may promote gephyrin phosphorylation. It would be interesting to understand how GABAergic synaptic and extrasynaptic receptor activity is orchestrated. Although it is known that different GABA_A_R subtypes modulate different aspects of adult neurogenesis, which downstream pathways are involved is still unclear.

Adult neurogenesis provides neuroblasts in a mature network. It is a unique feature that neuroblasts have to integrate into already existing network, lacking all the neurotrophic factors that are present during brain development. We still need to understand which mechanisms and which receptors GABA used to modulate the intracellular pathways that leads neuroblasts to integrate and mature. Beyond the fascinating quest to understand the mechanisms that drive adult neurogenesis, unraveling this issue will help us better understand not only brain function and development but also neurodevelopmental disorders. This may contribute to new strategies for cell replacement therapies.

### Conflict of interest statement

The authors declare that the research was conducted in the absence of any commercial or financial relationships that could be construed as a potential conflict of interest.
